# The association of polypoidal choroidal vasculopathy clinical phenotypes with previously reported genetic markers

**DOI:** 10.1007/s00417-020-04702-y

**Published:** 2020-04-23

**Authors:** Mingyue Luo, Xinyu Zhao, Jingyuan Yang, Youxin Chen

**Affiliations:** 1 Department of Ophthalmology, Peking Union Medical College Hospital, Chinese Academy of Medical Sciences, Beijing, China; 2 Key Laboratory of Ocular Fundus Diseases, Chinese Academy of Medical Sciences, Peking Union Medical College, Beijing, China

**Keywords:** Polypoidal choroidal vasculopathy, Single-nucleotide polymorphism, Genotype-phenotype association, Gene

## Abstract

**Purpose:**

Genetic studies have identified the association of some single-nucleotide polymorphisms (SNPs) with polypoidal choroidal vasculopathy (PCV), but little is known about whether these SNPs are related to PCV clinical features as well. We performed this study to examine the association of 12 SNPs with PCV clinical phenotypes.

**Methods:**

Sixty-nine PCV eyes of 69 patients were included. Genomic DNA was extracted from peripheral blood. Agilent SureSelect Human ALL Exon V6 was used to sequence the 12 SNPs previously reported to associate with PCV. Baseline best-corrected visual acuity (BCVA), sub-foveal choroidal thickness (SFCT), choroid maximum vascular diameter (MVD), choroidal vascular hyperpermeability (CVH), and greatest linear dimension (GLD) of entire lesion were measured and compared between patients of different genotypes. Fisher’s exact test and Mann-Whitney *U* test were mainly used to compare categorical variables and continuous variables respectively.

**Results:**

*HTRA1* rs2293870 was a protective factor of PCV or AMD in the fellow eye (*P* = 0.040) and was related with greater SFCT in PCV eye after multiple linear regression (*P* = 0.043). *C3* rs17030 was associated with smaller GLD (*P* = 0.033). *CFH* rs2274700 was related to lower MVD (*P* = 0.043) and was a protective factor for CVH (*P* = 0.034).

**Conclusion:**

Multiple PCV-associated SNPs are associated with PCV clinical phenotypes. The involvement of several synonymous SNPs calls for further research on the role of transcriptional alterations and trans-regulation of distant signaling pathways in PCV pathogenesis.

**Electronic supplementary material:**

The online version of this article (10.1007/s00417-020-04702-y) contains supplementary material, which is available to authorized users.

## Introduction

Polypoidal choroidal vasculopathy (PCV), first described by Yannuzzi et al. in the 1980s [1], is characterized by orange-red lesions on fundus examination and recurrent serosanguineous pigmented epithelium detachment (PED) [2]. While it remains controversial whether PCV is a subtype of neovascular age-related macular degeneration (nAMD) or a separate clinical entity due to their differences in epidemiology, clinical features, angiographic manifestations, and clinical courses [3], some consider PCV a part of pachychoroid-related disorders, which may represent different stages of a same pathogenic process [4].

Actually, PCV itself is diverse in clinical phenotypes. Until now, no universal classification could be applied to all studies. Many investigators stratified patients into polypoidal choroidal neovascularization (CNV) and typical PCV, depending on whether feeder vessels or draining vessels of polypoidal lesions are visible in ICGA [5]. Other criteria include but are not limited to sub-foveal choroidal thickness (SFCT) over 200 μm [6], BVN morphologies with or without leakage on FA [7], etc. Although the dissection of PCV into various clinical phenotypes helps predict disease course, treatment response, and prognosis, the variety of classification methods, the unpredictable transition between subtypes, and the inevitable impact of personal experience in imaging interpretation call for a more objective and subtle classification.

The diversity of classification reflects the unclarified nature of PCV. On the basis of genome-wide association studies (GWAS) of AMD, through examination of the association between genetic markers of AMD with PCV, various studies have proved PCV associated with genes related to inflammation, complement system, extra-cellular matrix homeostasis and lipid metabolism pathways, etc. [8]. Yet it should be highlighted that results of AMD genetic studies should be carefully testified before applied to PCV, since some AMD-associated SNPs are not associated with PCV, and although some are associated with both the disorders, the strength differed significantly [9]. Besides, the association of SNPs with PCV phenotypes is worth exploring. At present, the most studied SNP is *ARMS2* rs10490924, which is associated with ICGA-based classification.

Little is known about whether other SNPs are related to PCV clinical features as well. Thus, this study was performed to further examine the association of 12 PCV-associated SNPs with PCV clinical phenotypes.

## Materials and methods

Sixty-nine eyes of 69 patients diagnosed as PCV at Peking Union Medical College Hospital were enrolled in this study between February 2018 to April 2018 (21 females and 48 males with a mean age of 64.8 years). For bilaterally involved patients, only one eye was randomly chosen as study eye. Inclusion criteria were as follows: (1) clear diagnosis of PCV based on hyperfluorescent dilated polypoidal lesions (“polyps”) and/or BVN lesions on ICGA, especially within the first 6 min [10]; (2) treatment-naïve or a treatment-free period for at least 6 months; (3) patients who underwent comprehensive ophthalmic examination, including BCVA, slit-lamp examination, fundus photography, fluorescein angiography (FA), ICGA, and enhanced depth imaging (EDI) spectral-domain optical coherence tomography (SD-OCT). Written informed consent was obtained from all patients. The study was approved by the institutional review board of Peking Union Medical College Hospital and was conducted in accordance with the tenets of the Declaration of Helsinki.

General information was acquired through direct or telephone interview, including name, gender, age of disease-onset, comorbidities including hypertension and diabetes mellitus, conditions of contralateral eye, and baseline BCVA. BCVA was converted to the logarithm of the minimum angle of resolution (logMAR) equivalent.

SD-OCT images were obtained using Heidelberg Spectralis HRA-OCT device (HRA2, Heidelberg Engineering, Heidelberg, Germany). Specifically, EDI mode was selected to measure SFCT and choroid MVD (maximum vascular diameter). FA and ICGA were performed in the same session to measure GLD of entire lesion [11] and determine the angiographic type of each patient. The presence or absence of choroidal vascular hyperpermeability (CVH) was evaluated using ICGA images of middle and late phases [12]. All measurements were performed separately by three independent retinal specialists (ML, XZ, and JY), and any disagreement was discussed with a senior specialist (YXC). Average value for each continuous variable was calculated and used for statistical analysis.

### Sample collection, DNA genotyping, and SNP selection

Genomic DNA was extracted from peripheral blood using the Qiagen FlexiGene DNA Kit (Qiagen, Hilden, Germany) under standard procedures. Genotyping was performed using Agilent SureSelect Human ALL Exon V6 (Agilent Technologies, Santa Clara, CA).

Through literature reading, we selected 12 PCV-associated SNPs of 9 genes: rs10490924 [13] and rs2736911 [8] of *ARMS2*, rs1049331 [14] and rs2293870 [14] of *HTRA1*, rs2274700 [15] and rs1065489 [16] of *CFH*, rs547154 [17] of *C2*, rs541862 [17] of *CFB*, rs2217332 [18] of *HERPUD1*, rs5882 [19] of *CETP*, rs17030 [20] of *C3*, and rs78488639 [21] of *FPR1*. All aforementioned SNPs were reported to relate with PCV and are detectable with Agilent probes.

### Statistical analysis

Statistical analyses were performed using IBM SPSS Statistics version 22 (IBM Corp., Armonk, NY, USA). Categorical variables were compared between patients of different genotypes using Fisher’s exact test or binary logistic regression analysis. Mann-Whitney *U* test or one-way ANOVA was applied to compare continuous variables. Multiple linear regression was performed to assess the contributions of major risk factors to PCV phenotypes. Values of *P < 0.05* were considered statistically significant.

## Results

A total of 69 eyes of 69 PCV patients were included in the study. The characteristics of PCV patients, including gender, age, baseline BCVA, CVH, SFCT, comorbidities of hypertension and diabetes mellitus, and contralateral eye conditions, are summarized in Table 1. The features of selected SNPs, including located genes, base alteration, and distribution of genotypes, are summarized in Table 2.

**Table 1:** Characteristics of PCV patients

Characteristics	Results
Age of disease onset (y/o)	64.8 ± 7.45
Male/female, *n* (%)	48 (69.6%)/21 (30.4%)
Baseline BCVA (logMAR)	0.615 ± 0.395
CVH, *n* (%)	27 (39.5%)
SFCT, μm (SD)	259.3 (73.3)
Contralateral eye condition	
Healthy, *n* (%)	36 (52.2%)
PCV, *n* (%)	16 (23.2%)
AMD, *n* (%)	13 (18.8%)
Other diseases, *n* (%)	4 (5.8%)
Hypertension, *n* (%)	19 (27.5%)
Diabetes mellitus, *n* (%)	5 (7.2%)

*BCVA*, best-corrected visual acuity; *CVH*, choroidal vascular hyperpermeability; *SFCT*, sub-foveal choroidal thickness; *logMAR*, logarithm of minimal angle of resolution; *PCV*, polypoidal choroidal vasculopathy; *AMD*, age-related macular degeneration

**Table 2:** Distribution of genotypes of 69 patients

Gene	SNP	Base alteration	Minor homo	Hetero	Major homo
*ARMS2*	rs10490924	G→T	0.464	0.449	0.087
*ARMS2*	rs2736911	C→T	0.000	0.145	0.855
*HTRA1*	rs1049331	C→T	0.391	0.304	0.304
*HTRA1*	rs2293870	G→T	0.406	0.261	0.333
*CFH*	rs2274700	G→A	0.087	0.478	0.435
*CFH*	rs1065489	G→T	0.319	0.478	0.203
*C2*	rs547154	G→T	0.000	0.072	0.928
*CFB*	rs541862	T→C	0.000	0.072	0.928
*HERPUD1*	rs2217332	G→A	0.000	0.174	0.826
*CETP*	rs5882	G→A	0.217	0.464	0.319
*C3*	rs17030	G→A	0.246	0.609	0.145
*FPR1*	rs78488639	G→T	0.029	0.232	0.739

*ARMS2*, age-related maculopathy susceptibility 2 gene; *HTRA1*, HtrA serine peptidase 1 gene; *CFH*, complement factor H gene; *C2*, complement C2 gene; *CFB*, complement factor B gene; *HERPUD1*, homocysteine inducible ER protein with ubiquitin like domain 1 gene; *CETP*, cholesteryl ester transfer protein gene; *C3*, complement C3 gene; *FPR1*, formyl peptide receptor 1 gene; *SNP*, single-nucleotide polymorphism

Subjects whose genotype was TT or TG type of *HTRA1* rs2293870 were more likely to develop unilateral PCV with no AMD or PCV in the contralateral eye (*P* = 0.040, OR = 2.917, 95% CI = 1.028–8.273; Table 3). Further analysis using genotypic model showed that TT type was more likely to develop unilateral PCV than GG type (*P* = 0.021, OR = 3.958, 95% CI = 1.230–12.734), but there was no significant difference between TG type and GG type (*P* = 0.329). Rs2293870 was also associated with greater SFCT (*P* = 0.022). The average SFCT for TT and GT patients was 265.73 ± 66.74 μm, compared with 230.62 ± 82.93 μm for GG patients. *CFH* rs1065489 and *CETP* rs5882 were also related to greater SFCT, but only rs2293870 remained significantly associated after multiple linear regression (*P* = 0.047).

**Table 3:** SNPs associated PCV clinical phenotypes (categorical variables)

Clinical phenotype	Gene	SNP	SNP influence	OR (95%CI)	*P*
Contralateral eye healthy ^a^	*HTRA1*	rs2293870	Increase	2.917 (1.028–8.273)	0.040
CVH^b^	*CFH*	rs2274700	Decrease	0.344 (0.126–0.935)	0.034

^a^Contralateral eye

^b^PCV eye

*OR*, odds ratio; *CI*, confidence interval; *SNP*, single-nucleotide polymorphism; *HTRA1*, HtrA serine peptidase 1 gene; *CVH*, choroidal vascular hyperpermeability; *CFH*, complement factor H gene

MVD was associated with the minor allele of *CFH* rs2274700 (*P* = 0.043; Table 4). The average MVD of the 39 subjects who had at least one copy of minor allele (AA and AG) was 183.85 ± 52.10 μm, compared with 207.87 ± 58.04 μm for the 30 subjects homogeneous for the major allele (GG) (*P* = 0.043). No significant variation was detected using genotypic model. *CFH* rs2274700 was also identified as a protective factor from CVH (*P* = 0.034, OR = 0.344, 95% CI = 0.126–0.935; Table 3).

**Table 4:** SNPs associated PCV clinical phenotypes (continuous variables)

Clinical phenotypes	Gene	SNP	Genotype	Number of patients	Mean value	Mann-Whitney *U* test
SFCT	*HTRA1*	rs2293870	TT or GT	46	265.73 ± 66.74	0.022
			GG	23	230.62 ± 82.93	
	*CFH*	rs1065489	TT or TG	55	262.96 ± 74.34	0.037
			GG	14	218.95 ± 62.39	
	*CETP*	rs5882	AA or AG	47	266.53 ± 76.36	0.047
			GG	22	227.30 ± 61.49	
MVD	*CFH*	rs2274700	AA or AG	39	183.85 ± 52.10	0.043
			GG	30	207.87 ± 58.04	
GLD	*C3*	rs17030	AA or AG	59	4909.63 ± 2694.85	0.033
			GG	10	7558.00 ± 1291.37	

*SNP*, single-nucleotide polymorphism; *SFCT*, sub-foveal choroidal thickness; *MVD*, choroid maximum vascular diameter; *GLD*, greatest linear dimension; *HTRA1*, HtrA serine peptidase 1 gene; *CFH*, complement factor H gene; *CETP*, cholesteryl ester transfer protein gene; *C3*, complement C3 gene

GLD was related with *C3* rs17030 (*P* = 0.033; Table 4). Fifty-nine subjects had at least one copy of the minor allele (AA and AG). The average GLD of these subjects was 4909.63 ± 2694.85 μm. Ten subjects were homogeneous for the major allele (GG). The average GLD was 7558.00 ± 1291.37 μm. Further one-way ANOVA analysis showed that GLD of both AA and AG type was significantly smaller than GG type (4817.94 ± 2685.73, 4946.74 ± 2730.13 μm). Thus, *C3* rs17030 is a protective factor from larger GLD.

No association was detected between the other SNPs and PCV clinical phenotypes in this study.

## Discussion

In this study, we found that subjects with variants of *HTRA1* rs2293870 were more likely to develop unilateral PCV. Rs2293870, rs5882, and rs1065489 were all associated with larger SFCT in this study, but only rs2293870 remained significantly associated after logistic regression with age. Rs2293870 is a synonymous mutation (c.108G>T) of the first exon of *HTRA1*, resulting in altered mRNA secondary structure, but no difference in expressional level and protein sequence [22]. Yet some studies indicated the difference of protein structure, which renders varied properties of heat-induced unfolding, trypsin accessibility, and secretion behavior [23]. Direct binding and proteolysis of transforming growth factor β1 (TGF-β1) is seen only in normal HTRA1, but not the c.108G>T isomer, which results in varied downstream TGF-β1 pathway activities. Yet it remains to be clarified how these changes in mRNA and protein secondary structure are related with PCV pathogenesis, disease laterality, and SFCT.

Our study first reported rs2274700 as a protective factor for CVH. Rs2274700 is a synonymous SNP of the tenth exon of *CFH*. Multiple studies have reported the association of rs2274700 with PCV, but the association seems stronger with AMD. Li et al. [24] examined 84 polymorphisms in and around *CFH* in AMD patients and found that the disease susceptibility was related more to certain haplotypes in a specific 32 kb region instead of SNPs. As a synonymous SNP, although the secondary structure of mRNA and CFH may slightly differ, but since rs2274700 is in linkage disequilibrium with multiple SNPs and copy number variations (CNP) in this region, it is not sound to attribute the disease susceptibility to rs2274700 itself. These variations may synergistically regulate the expression of *CFH* or other nearby complement genes [24]. Further detailed, in-depth genetic analysis should be carried out to dissect the association of disease susceptibility with this region.

In this study, we have proved that some of previously reported PCV-associated SNPs are also related to disease phenotypes. We first reported *HTRA1* rs2293870 to be related with greater SFCT, and a protective factor for fellow eye involvement. Our results also showed that *C3* rs17030 is associated with smaller GLD and *CFH* rs2274700 is a protective factor for CVH. These findings above indicate molecular interactions of these SNP with PCV clinical phenotypes. Although some SNPs are within intron region, transcriptional and translational alterations may occur and so does trans-regulation of distant signaling pathways. Limitations of this study include a relatively small sample size and inevitable selection bias, calling for replication studies with larger sample size and subjects from various genetic backgrounds.

In conclusions, multiple PCV-associated SNPs were associated with PCV clinical phenotypes. Further research into the molecular interactions of these key SNPs may provide valuable hints for PCV pathogenesis.

## Electronic supplementary material

ESM 1 (DOCX 73 kb)
