# Supplementary material for: The association of polypoidal choroidal vasculopathy clinical phenotypes with previously reported genetic markers
Source: Graefes Arch Clin Exp Ophthalmol. 2020 Apr 23;258(6):1199–203. doi: 10.1007/s00417-020-04702-y (PMC7237508; doi:10.1007/s00417-020-04702-y)
Supplement: Supplementary file 1 — (DOCX 73 kb) [file 417_2020_4702_MOESM1_ESM.docx]

|  | **unilateral PCV** | **MVD** | **CVH** | **GLD** | **SFCT** |
| --- | --- | --- | --- | --- | --- |
| **rs10490924** | 0.201 | 0.219 | 0.392 | 0.525 | 0.079 |
| **rs2736911** | 0.311 | 0.204 | 0.051 | 0.306 | 0.109 |
| **rs1049331** | 1.000 | 0.084 | 0.440 | 0.593 | 0.052 |
| **rs2293870** | ***0.040**** | 0.154 | 1.000 | 0.990 | ***0.022**** |
| **rs2274700** | 1.000 | ***0.043**** | ***0.034**** | 0.952 | 0.937 |
| **rs1065489** | 0.769 | 0.182 | 1.000 | 0.483 | ***0.037**** |
| **rs547154** | 0.359 | 0.450 | 0.389 | 0.104 | 0.796 |
| **rs541862** | 0.359 | 0.450 | 0.389 | 0.104 | 0.796 |
| **rs2217332** | 1.000 | 0.831 | 0.527 | 0.692 | 0.646 |
| **rs5882** | 0.452 | 0.357 | 0.064 | 0.847 | ***0.047**** |
| **rs17030** | 0.502 | 0.357 | 1.000 | ***0.033**** | 0.229 |
| **rs78488639** | 0.789 | 0.197 | 0.052 | 0.140 | 0.408 |
| SNP, single-nucleotide polymorphism; PCV, polypoidal choroidal vasculopathy; MVD, maximum vascular diameter; CVH, choroidal vascular hyperpermeability; GLD, great linear dimension; SFCT, sub-foveal choroidal thickness  *Statistically significant *P* value. | | | | | |

**Supplementary Table 1 All P values of the association of SNPs and PCV clinical phenotypes (dominant model)**
